# Genetic variants in FGFR2 and FGFR4 genes and skin cancer risk in the Nurses' Health Study

**DOI:** 10.1186/1471-2407-9-172

**Published:** 2009-06-06

**Authors:** Hongmei Nan, Abrar A Qureshi, David J Hunter, Jiali Han

**Affiliations:** 1 Program in Molecular and Genetic Epidemiology, Department of Epidemiology, Harvard School of Public Health, Boston, MA 02115, USA; 2 Channing Laboratory, Department of Medicine, Brigham and Women's Hospital and Harvard Medical School, Boston, MA 02115, USA; 3 Department of Dermatology, Brigham and Women's Hospital and Harvard Medical School, Boston, MA 02115, USA

## Abstract

**Background:**

The human fibroblast growth factor (FGF) and its receptor (FGFR) play an important role in tumorigenesis. Deregulation of the *FGFR2 *gene has been identified in a number of cancer sites. Overexpression of the *FGFR4 *protein has been linked to cutaneous melanoma progression. Previous studies reported associations between genetic variants in the *FGFR2 *and *FGFR4 *genes and development of various cancers.

**Methods:**

We evaluated the associations of four genetic variants in the *FGFR2 *gene highly related to breast cancer risk and the three common tag-SNPs in the *FGFR4 *gene with skin cancer risk in a nested case-control study of Caucasians within the Nurses' Health Study (NHS) among 218 melanoma cases, 285 squamous cell carcinoma (SCC) cases, 300 basal cell carcinoma (BCC) cases, and 870 controls.

**Results:**

We found no evidence for associations between these seven genetic variants and the risks of melanoma and nonmelanocytic skin cancer.

**Conclusion:**

Given the power of this study, we did not detect any contribution of genetic variants in the *FGFR2 *or *FGFR4 *genes to inherited predisposition to skin cancer among Caucasian women.

## Background

The human fibroblast growth factor (FGF) and its receptor families consist of 22 structurally related FGF members and four high-affinity tyrosine kinase FGF receptors (FGFR1 to 4) [1,2]. The four FGFRs generate ligand-binding specific isoforms by tissue-specific alternative mRNA splicing of the genes [3-7]. FGFs and their receptors have an important role in cell signaling [8]. The formation of the FGF-FGFR complex activates the intracellular tyrosine kinase, which mediates signal transduction through the direct phosphorylation of adaptor proteins [9]. These complex FGF signaling networks are crucial in the multiple cell biological activities, such as proliferation, differentiation, mitogenesis, migration, and apoptosis, and are thus implicated in tumorigenesis [10-12].

The *FGFR2*, known as a unique high-affinity receptor for keratinocyte growth factor (KGF or FGF7), is expressed in the keratinocytes of the skin epidermis, hair follicles, and mesenchymal tissues [5,13,14]. An experiment in transgenic mice with *FGFR2 *mutation in the keratinocyte showed that normal signal transduction was blocked by binding of its ligand KGF [15]. It has been reported that the *FGFR2 *plays a role in tumor suppression in the skin [16]. In addition, the increased *FGFR2 *gene expression has been related to the genetic variants in intron 2 of the *FGFR2 *gene [17] and deregulation of *FGFR2 *gene expression and/or gene mutation has been identified in various kinds of human cancers, such as breast, prostate, endometrial, colon, bladder, and thyroid cancers [17-22]. Recently, two genome-wide association studies have identified some genetic variants in the *FGFR2 *gene that were highly associated with breast cancer [23,24].

The *FGFR4 *gene located on the chromosome 5 spans approximately 11.3 kb and is composed of 18 exons [25]. Overexpression of the FGFR4 protein has been associated with cutaneous melanoma progression [26]. High expression of FGFR4 has also been observed in breast cancer, prostate cancer, pancreatic cancer, and renal cell carcinoma [27-30]. Furthermore, SNP rs351855 located in exon 9 of the *FGFR4 *gene results in an amino acid change (Gly388Arg) in the transmembrane domain of the receptor and has been associated with tumor progression in, for example, cutaneous nodular malignant melanoma, breast cancer, lung adenocarcinoma, prostate cancer, and head and neck cancer [26,31-36].

We conducted a nested case-control study of Caucasians within the Nurses' Health Study (NHS) to evaluate whether the four breast cancer-related SNPs in the *FGFR2 *gene (rs11200014, rs2981579, rs1219648, and rs2420946) [24] and the three common variants (tag-SNPs) in the *FGFR4 *gene (rs1966265, rs376618, and rs351855) are associated with the risk of three skin cancer types including melanoma, squamous cell carcinoma (SCC), and basal cell carcinoma (BCC).

## Methods

Eligible cases in this study consisted of women with incident skin cancer from the subcohort of the NHS who gave a blood specimen in 1989–1990 (*n *= 32,826), including SCC and BCC cases with a diagnosis any time after blood collection up to June 1, 1998 and melanoma cases up to June 1, 2000 with no previously diagnosed skin cancer. A common control series was randomly selected from participants who gave a blood sample and were free of diagnosed skin cancer up to and including the questionnaire cycle during which the case was diagnosed. One or two controls were matched to each case by year of birth (± 1 year). All subjects were drawn from the U.S. non-Hispanic Caucasian women in this study. The nested case-control study consisted of 218 incident melanoma cases, 285 incident SCC cases, a sample of 300 BCC cases from the large number of incident cases, and 870 age-matched controls. The informed consent was obtained from the participants in this study. The study protocol was approved by the Committee on Use of Human Subjects of the Brigham and Women's Hospital, Boston, MA.

We obtained information regarding skin cancer risk factors from the prospective biennial questionnaires and a retrospective supplementary questionnaire. Information on natural hair color at age 20 and childhood and adolescent tanning tendency were collected in the 1982 prospective questionnaire. Ethnic group was ascertained in the 1992 questionnaire. In the skin cancer nested case-control study, natural skin color and other sun exposure-related information were collected by the retrospective supplementary questionnaire in 2002. The response rates of cases and controls were 92% and 89%, respectively. A cumulative lifetime sun exposure while wearing a bathing suit for each individual was developed by combining the UV database and the information obtained from the supplementary questionnaires. We constructed a multivariate confounder score to create a constitutional susceptibility score [37], summarizing natural skin color, natural hair color, child or adolescent tendency to burn, and the number of palpably raised moles on arms. We used this score to define women with constitutional susceptibility [38]. In addition, the 11 states of residence of cohort members at baseline were grouped into three regions: Northeast (Connecticut, Massachusetts, Maryland, New Jersey, New York, and Pennsylvania), Northcentral (Michigan and Ohio), and West and South (California, Texas, and Florida).

Information on the seven SNPs in the *FGFR2 *and *FGFR4 *genes is presented in Table 1. Four SNPs in intron 2 of the *FGFR2 *gene (rs11200014, rs2981579, rs1219648, and rs2420946) genotyped in this study were breast cancer-related SNPs identified by a recent genome-wide association study conducted by our group [24]. For the *FGFR4 *gene, based on the HapMap phase II SNP genotype data, we chose three tag-SNPs (rs1966265, rs376618, and rs351855) as surrogates for untyped polymorphisms in the *FGFR4 *gene using the HapMap Project 90 (30 trios) Caucasian samples from a US Utah population with Northern and Western European ancestry collected in 1980 by the Centre d'Etude du Polymorphisme Humain (CEPH) [39]. Briefly, the tag-SNPs (minor allele frequency > 0.05) were selected using the Tagger program of (r^2^>0.8), which combines the simplicity of pairwise r^2 ^methods [40] with the potential efficiency of multimarker haplotype approaches [41].

**Table 1:** Seven SNPs in the *FGFR2 *and *FGFR4 *genes

SNP	rs#	Chromosome	Location	MAF-controls (%)^a^	MAF-CEU (%)^b^
*FGFR2 *intron 2	rs11200014	10	123324920	42	47
*FGFR2 *intron 2	rs2981579	10	123327325	42	47
*FGFR2 *intron 2	rs1219648	10	123336180	40	47
*FGFR2 *intron 2	rs2420946	10	123341314	40	47
*FGFR4 *Val10Ile	rs1966265*	5	176449237	-	20
	rs12519145*	5	176488129	22	19
*FGFR4 *Leu136Pro	rs376618	5	176450403	24	26
*FGFR4 *Gly388Arg	rs351855	5	176452849	31	28

*The SNP rs1966265 failed the assay and the rs12519145 was genotyped instead (r^2 ^= 0.8).

^a ^Minor allele frequency (MAF) was calculated among controls in this study.

^b ^MAF was based on the HapMap CEU (Utah residents with ancestry from northern and western Europe) samples.

We genotyped these seven SNPs by the 5' nuclease assay (TaqMan^®^) in 384-well format, using the ABI PRISM 7900 HT Sequence Detection System (Applied Biosystems, Foster City, CA). TaqMan^® ^primers and probes were designed with the Primer Express^® ^Oligo Design software v2.0 (ABI PRISM). Due to assay failure, we genotyped rs12519145 as a surrogate for the *FGFR4 *rs1966265 (r^2 ^= 0.8). Laboratory personnel were blinded to case-control status, and 10% blinded quality control samples (duplicate samples) were inserted to validate genotyping procedures; concordance for the blinded quality control samples was 100%. Primers, probes, and conditions for genotyping assays are available upon request.

We used the χ^2 ^test to assess whether the genotypes for all seven SNPs were in Hardy-Weinberg equilibrium among the controls. We compared each type of skin cancer with the common control series to increase the statistical power. We evaluated the association between each genotype and skin cancer risk using unconditional logistic regression. An additive model was used to calculate the *p*-value on skin cancer risk according to an ordinal coding for genotype (0, 1 or 2 copies of SNP minor allele). For the four *FGFR2 *SNPs and three *FGFR4 *SNPs, haplotype frequencies and expected haplotype counts for each individual were estimated using a simple expectation-maximization algorithm, as implemented in SAS PROC HAPLOTYPE. The analyses of the associations between haplotypes and skin cancer risk were performed using the expectation-substitution technique [42]. All statistical analyses were two-sided and carried out using SAS V9.1 (SAS Institute, Cary, NC).

The Quanto statistical software version 1.2.3 was used for power calculation [43]. We calculated the power to detect the specified ORs at various allele frequencies of variant allele in additive models. The calculations were based on a two-sided alpha of 0.05. For melanoma (SCC or BCC), we have 80% power to detect an OR of 1.80 (1.72 or 1.70), 1.48 (1.42 or 1.41), and 1.35 (1.32 or 1.31) if the minor allele frequency is 5%, 15%, and 40%, respectively.

## Results and discussion

A detailed description of the characteristics of cases and controls in the skin cancer nested case-control study has been provided previously [44]. In brief, at the beginning of the follow-up of this nested case-control study, the nurses were between 43 and 68 years old (mean age, 58.7 years). The mean ages at diagnosis for incident melanoma, SCC, and BCC cases were 63.4, 64.7, and 64.0 years, respectively. A family history of skin cancer was a risk factor for all three types of skin cancer. Skin cancer cases had lighter pigmentation (skin color and hair color), more moles on the arms, higher cumulative sun exposure while wearing a bathing suit, and more lifetime severe sunburns that blistered than controls.

The genotype distributions of the seven SNPs evaluated in this study were in Hardy-Weinberg equilibrium among controls. The minor allele frequencies of these seven SNPs among controls in this study were similar to those from HapMap CEU data. We evaluated the main effect of each polymorphism across three types of skin cancer (Table 2) and observed no significant associations between these seven SNPs and skin cancer risk. The multivariate analyses controlling for age and skin cancer risk factors showed results similar to the age-adjusted analyses (Additional file 1). Furthermore, we performed a global test to evaluate the difference in *FGFR2 *and *FGFR4 *haplotype frequencies between cases and controls (Table 3) and found no significant associations with skin cancer risk, which was consistent with the results of the single SNP analyses presented in Table 2.

**Table 2:** Associations between the seven SNPs in the *FGFR2 *and *FGFR4 *genes and skin cancer risk

SNP	Melanoma		SCC		BCC	
	
	Additive OR*	*p *for trend	Additive OR*	*p *for trend	Additive OR*	*p *for trend
*FGFR2 *rs11200014	0.95 (0.77–1.19)	0.67	0.90 (0.74–1.10)	0.30	1.03 (0.85–1.26)	0.73
*FGFR2 *rs2981579	0.96 (0.77–1.19)	0.70	0.92 (0.75–1.12)	0.40	1.11 (0.91–1.36)	0.29
*FGFR2 *rs1219648	0.96 (0.77–1.20)	0.75	0.87 (0.71–1.07)	0.18	1.06 (0.87–1.29)	0.57
*FGFR2 *rs2420946	1.08 (0.85–1.38)	0.53	0.89 (0.72–1.10)	0.28	0.99 (0.81–1.21)	0.91
*FGFR4 *rs1966265**	1.16 (0.90–1.48)	0.26	1.00 (0.79–1.26)	1.00	0.94 (0.74–1.19)	0.61
*FGFR4 *rs376618	0.88 (0.67–1.14)	0.33	1.04 (0.83–1.31)	0.73	0.87 (0.69–1.11)	0.27
*FGFR4 *rs351855	1.09 (0.87–1.38)	0.44	0.90 (0.73–1.12)	0.35	1.13 (0.93–1.39)	0.21

*Unconditional logistic regression adjusted for age.

**The SNP rs1966265 failed the assay and the rs12519145 was genotyped instead (r^2 ^= 0.8).

**Table 3:** Haplotypes for the SNPs in the *FGFR2 *and *FGFR4 *genes and skin cancer risk

*FGFR2*						Melanoma		SCC		BCC	
				Controls		Cases		Cases		Cases	

A	B	C	D	n	%	n	%	n	%	n	%

0	0	0	0	779	56.4	166	55.3	286	59.8	270	55.1
				Multivariate OR		1.00		1.00		1.00	
1	1	1	1	532	38.5	118	39.3	177	37.0	196	40.0
				Multivariate OR		1.06 (0.82–1.38)		0.90 (0.72–1.11)		1.06 (0.85–1.32)	
1	1	0	0	25	1.8	3	1.0	8	1.7	8	1.6
				Multivariate OR		0.55 (0.16–1.89)		0.89 (0.39–2.00)		0.90 (0.40–2.05)	
1	1	1	0	17	1.2	8	2.7	3	0.6	6	1.2
				Multivariate OR		2.42 (1.00–5.87)		0.46 (0.13–1.60)		1.03 (0.40–2.69)	
Rare < 1%				29	2.1	5	1.7	4	0.8	10	2.1
				Multivariate OR		0.80 (0.31–2.06)		0.41 (0.15–1.13)		0.98 (0.50–1.93)	

A: rs11200014; B: rs2981579; C: rs1219648; D: rs2420946											

	***FGFR4***					**Melanoma**		**SCC**		**BCC**	

				Controls		Cases		Cases		Cases	

	A	B	C	n	%	n	%	n	%	n	%

	0	0	1	446	29.7	118	30.7	121	25.8	164	32.2
				Multivariate OR		1.00		1.00		1.00	
	0	0	0	391	26.0	89	23.2	130	27.7	133	26.1
				Multivariate OR		0.86 (0.63–1.17)		1.22 (0.92–1.62)		0.93 (0.71–1.21)	
	0	1	0	343	22.8	83	21.7	113	24.0	106	20.9
				Multivariate OR		0.91 (0.66–1.26)		1.21 (0.90–1.64)		0.82 (0.62–1.10)	
	1	0	0	293	19.5	84	22.0	95	20.3	95	18.8
				Multivariate OR		1.08 (0.78–1.49)		1.19 (0.88–1.62)		0.88 (0.66–1.18)	
	Rare < 1%			30	2.0	9	2.4	10	2.2	10	1.9
				Multivariate OR		1.21 (0.49–2.96)		1.31 (0.55–3.10)		0.89 (0.37–2.16)	

A: rs1966265*; B: rs376618; C: rs351855											

0, common allele; 1, rare allele.

Logistic regression adjusted for age.

*p*-values for global tests are >0.05.

*The SNP rs1966265 failed the assay and the rs12519145 was genotyped instead (r^2 ^= 0.8).

The potential contribution of the FGF/FGFR family to the development of skin cancer has been suggested. For example, the basic FGF (bFGF) alternatively named *FGF2 *binds to distinct splice variants of the four FGFRs and acts as a potent activator in the proliferation and differentiation of melanocytes [45]. It has been noted that the combination of bFGF with ultraviolet (UV) light, the main risk factor for skin cancer, may lead to cutaneous melanoma induction [46]. In this study, we assessed the associations between the genetic variants in the *FGFR2 *and *FGFR4 *genes and the three types of skin cancer simultaneously with a modest sample size in each cancer type. Only one study has attempted to assess the relation of the *FGFR4 *Gly388Arg with the progression of melanoma in melanoma patients, and observed that the *FGFR4 *Arg388 allele was associated with tumor thickness and nodular malignant melanoma [26]. We did not observe a significant association of this allele with skin cancer risk. It seems that this SNP acts as a potential marker for the progression of skin cancer rather than susceptibility to skin cancer. Spinola et al. reported similar results for lung adenocarcinoma, i.e., that this allele revealed association with progression of cancer but a lack of association with the risk of cancer [33]. *FGFR2 *possesses the largest genomic structure among the FGFR family, with at least 22 exons and 21 introns and has been implicated in distinct types of cancer [47]. Also, recent *in vitro *and *in vivo *studies showed that loss-of-function *FGFR2 *mutations occur in a subset of melanomas [48]. It would be important to comprehensively examine the association of the common genetic variants in the entire *FGFR2 *gene region with skin cancer risk.

## Conclusion

In conclusion, we did not detect any contribution of genetic variants in the *FGFR2 *or *FGFR4 *genes to inherited predisposition to skin cancer among Caucasian women.

## List of Abbreviations

**FGFR**: Fibroblast Growth Factor Receptor; **BCC**: Basal Cell Carcinoma; **SCC**: Squamous Cell Carcinoma; **OR**: Odds Ratio; **CI**: Confidence Interval; **UV**: Ultraviolet.

## Competing interests

The authors declare that they have no competing interests.

## Authors' contributions

All authors have contributed to designing the study and analyzing and interpreting the data, as well as to the writing of the manuscript. All authors have read and approved this manuscript.

## Pre-publication history

The pre-publication history for this paper can be accessed here:

http://www.biomedcentral.com/1471-2407/9/172/prepub

## Supplementary Material

Additional File 1 **Supplementary Table S1. Associations between the seven SNPs in the *FGFR2 *and *FGFR4 *genes and skin cancer risk**. The data provided represent the results of the associations between seven SNPs in the FGFR2 and FGFR4 genes and skin cancer risk. Click here for file
